# Isolated Pre-existing HLA-DP Donor-Specific Antibodies are Associated With Poorer Outcomes in Renal Transplantation

**DOI:** 10.1016/j.ekir.2022.07.014

**Published:** 2022-08-03

**Authors:** Adrienne Seitz, Katherine Mounsey, Pamela Hughes, Katherine Cullen, Matthew Welberry Smith, Sunil Daga, Clive Carter, Brendan Clark, Richard Baker

**Affiliations:** 1 Renal Transplant Unit, St James’s University Hospital, Leeds, UK; 2 Transplant Immunology, St James’s University Hospital, Leeds, UK

**Keywords:** antibody-mediated rejection, donor-specific antibodies, HLA-DP

## Abstract

**Introduction:**

The importance of donor-specific antibodies (DSAs) in renal transplantation has long been recognized, but the significance of human leukocyte antigen (HLA)-DP antibodies remains less clear. We performed a retrospective single center study of renal transplants with pre-existing isolated HLA-DP-DSAs to assess clinical outcomes.

**Methods:**

Twenty-three patients with isolated HLA-DP-DSAs were compared with 3 control groups as follows: standard immunological risk (calculated reaction frequency [cRF] < 85%, no current or historical DSA, no repeat mismatched antigens with previous transplants, *n* = 46), highly sensitized (cRF > 85%, *n* = 27), and patients with HLA-DP antibodies that were not donor-specific (*n* = 18). Univariate and multivariate analyses were performed comparing antibody-mediated rejection (ABMR)-free and graft survival. Factors in the final multivariable models included patient group, % cRF, B-cell flow crossmatch (BFXM) positivity and regrafts.

**Results:**

Over a median follow-up of 1197 days, 65% of HLA-DP-DSA patients had ABMR on indication biopsies, and 30% of HLA-DP-DSA patients lost their graft. Pre-existing HLA-DP DSAs remained the single factor associated with ABMR after multivariable analysis (hazard ratio [HR] = 9.578, *P* = 0.012). Patients with HLA-DP DSAs had increased microvascular scores (*P* = 0.0346) and worse transplant glomerulopathy (*P* = 0.015) on biopsy compared with the standard immunological risk group. Furthermore, flow crossmatch (FXM) positivity did not help inform on the risk of graft failure or ABMR in patients with preformed DP-DSA.

**Conclusion:**

Transplants with pre-existing HLA-DP-DSAs should be considered high risk. Routine laboratory tests are unable to further risk stratify these patients. Recipients should be considered for intensified immunosuppression and closely monitored.

## Graphical abstract

**Figure:**
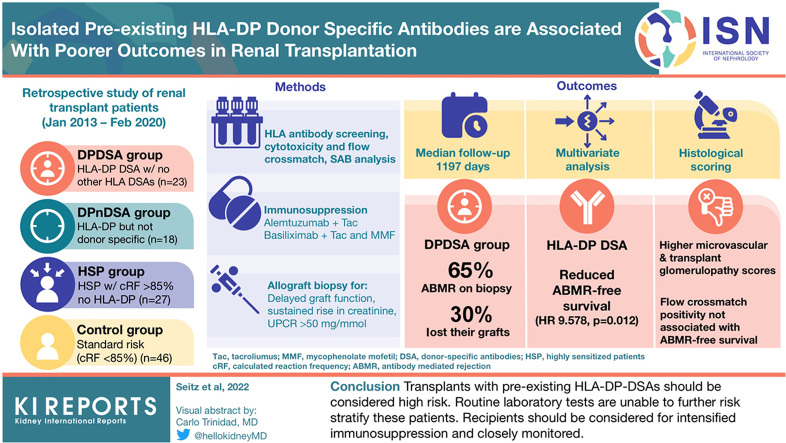

The importance of DSAs in renal transplantation has long been recognized and led to the establishment of the pretransplantation crossmatch.^1^ Understanding of the pathogenesis of antibody-mediated damage to renal allografts has increased over the last 3 decades as a result of technological advances in both the detection of antibodies and their associated injury pathways.^2^^,^^3^ As increased sensitivity and improved definition of antibody analysis has become available, scrutiny has turned to the relative importance of different antibodies contributing to graft injury.

Antibody-mediated damage of renal allografts is recognized as a major cause of graft loss and the role of human leukocyte antigen (HLA) class II antibodies has been increasingly acknowledged.^4^ Whereas renal endothelial cells may not express class II HLA constitutively, they have been demonstrated to express these molecules following inflammatory stimuli.^5^^,^^6^ The subsequent development of class II antibodies may lead to acute ABMR or more insidious chronic ABMR, which is associated with the development of transplant glomerulopathy. Although attention was initially focused on antibodies against HLA-DR it has been increasingly recognized that HLA-DQ and HLA-DP antibodies are also important.^4^

Early data suggested that performing transplants in the presence of HLA-DP antibodies was not detrimental to graft outcomes, although no distinction was made between DSA and non-DSA.^7^ A study of 4900 cadaveric renal transplants showed that whereas HLA-DP mismatch was not associated with a deleterious effect in first transplant recipients, each HLA-DP mismatch was associated with a step-wise reduction in 1-year graft survival rates for retransplants. This was particularly significant in sensitized recipients with 50% reactivity of preformed lymphocytotoxic antibodies.^5^ HLA-DPB mismatches at the epitope level were also associated with reduced graft survival in retransplants only, suggesting that HLA-DP antibodies may be a contributing factor.^6^ More recently, several case reports have demonstrated the pathogenicity of preformed HLA-DP DSAs identified by FXM,^8^^,^^9^ or solid phase assays alone.^10^ “Third party” HLA-DP antibodies with cross-reactive epitopes have also been implicated in chronic ABMR.^11^

We performed a retrospective study of our experience with HLA-DP antibody incompatible renal transplants as defined by single antigen beads (SABs). In this group, there was no T-cell positivity in the FXM, and B-cell positivity occurred in 32% of patients.

## Methods

### Patient Selection

**Figure 1:**
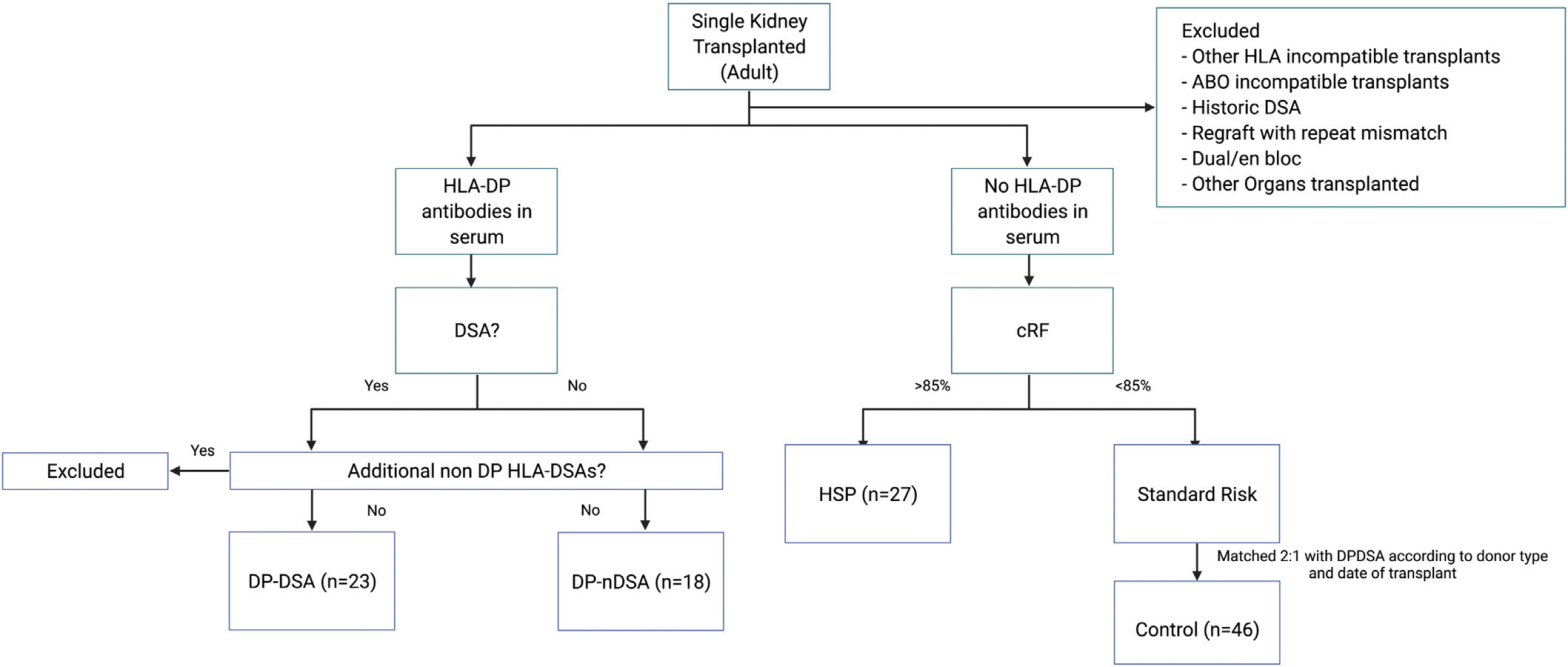
Study design. cRF, calculated reaction frequency; DSA, donor-specific antibodies; HLA, human leukocyte antigen; HSP, highly sensitized patients. Created using Biorender.com.

In this retrospective study, the time of offer (TOO) or current sera were assessed in all adult renal transplant recipients who were transplanted between January 2013 and February 2020. The group of patients with pre-existing HLA-DP DSAs in the absence of other HLA-DSAs formed the primary study group (DPDSA). The DPDSA cohort was compared with 3 other groups. The first group included patients who had HLA-DP antibodies that were not donor-specific (DPnDSA group). The second group included highly sensitized patients (HSP) with a cRF (see Supplementary Figure S1) greater than 85% but who had no HLA-DP antibodies in the TOO sera (HSP group). The third group (control group) were standard immunological risk recipients (with cRF < 85%) who received contemporaneous transplants that were matched in a 2:1 ratio with the DPDSA cohort according to donor type. This was to account for changes in both the local crossmatching (removal of the complement dependent cytotoxicity [CDC] crossmatch) and national UK allocation policies that occurred during the study period. Patients were excluded from the analysis if there was a historical HLA-DSA which was not present in the TOO sample. This was to limit adverse outcomes that could be attributed to anamnestic B-cell responses. Similarly, regrafts with HLA mismatches that repeated mismatches of previous transplants were excluded. ABO-incompatible and all other HLA antibody incompatible transplants were excluded. The study design is demonstrated in Figure 1.

Patients had HLA typing in line with requirements for the UK allocation scheme (the minimum requirement included HLA-A, B, C, DRB1, DRB3/4/5, and DQB1 until September 2019. After September 2019, the requirement expanded to include HLA-DQA1, DPB1, and DPA1).^12^^,^^13^ For DPDSA patients, the HLA-DPB1 and HLA-DPA1 types were confirmed for both donors and recipients using reverse sequence specific oligonucleotides (LABType) to allow differentiation between DPA1 and DPB1 DSAs (Donor DPA1–15/23, donor DPB1 – 23/23, recipient DPA1 19/23, and recipient DPB1 22/23).

### HLA Antibody Screening and Histocompatibility Testing

Wait-list patients were screened quarterly using LABScreen mixed beads (ONE LAMBDA, Canoga Park, CA). If positive, the specificities were characterized using LABScreen Single Antigen beads (ONE LAMBDA, SAB) according to manufacturer instructions. To overcome the prozone effect, sera were pretreated with 6% ethylenediaminetetraacetic acid to achieve a 1:50 ethylenediaminetetraacetic acid:serum dilution. Our protocol for reporting HLA antibodies included a positivity threshold of normalized median fluorescent intensity (MFI) more than 2000 combined with a ratio score of 6 or more after correcting for nonspecific binding to the negative control bead (Supplementary Figure S2). If the antibody specificity was represented by more than 1 bead, and all beads were positive, an average MFI was calculated for the antibody. If only some of the beads were positive, the represented allelic antibodies were reported with the average MFI obtained for each positive allelic antibody. If more than 1 HLA-DP DSA was detected, the MFIs obtained from each DSA were added together, and this cumulative MFI was included in the analysis. The TOO sera from the DPDSA group (21 out of 23) were retested using the locally validated LifeCodes Single Antigen microbead assay (Immucor, Norcross, GA) to confirm the presence of DPDSAs.^14^

A combination of CDC crossmatch, FXM and SAB analysis were used to determine histocompatibility and risk of proceeding with the transplant. Transplants were progressed in the case of “technical” positive wet crossmatches if the reactivity could not be attributed to the presence of a donor-relevant HLA antibody. If DSAs were identified pretransplant, the decision to progress was made based on individual patient history and risk appetite. As donor HLA-DP typing was not required for organ allocation, it was not always apparent that an HLA-DP incompatible offer had been received until after the transplant had occurred. This was usually known by the next working day following TOO serum testing or after the donor HLA-DP typing was performed locally.

### Donor HLA-DPB1 Expression Levels

Two single nucleotide polymorphism variants (rs9277534G and rs2281389A/G) that are present in the 3′ untranslated region of HLA-DPB1 have been shown to be associated with differing HLA-DPB1 transcript levels.^15^^,^^16^ These single nucleotide polymorphisms have been described to be in linkage disequilibrium with certain HLA-DPB1 alleles in Caucasians.^15^ DPDSA patients were categorized into low and high expression groups inferred from the donor HLA-DPB1 alleles.

### Routine Immunosuppression

Standard immunosuppression consisted of alemtuzumab induction with tacrolimus monotherapy, or basiliximab induction with tacrolimus and mycophenolate mofetil as previously described.^17^ Mycophenolate mofetil was routinely added if there were 2 HLA-DR mismatches between donor and recipient. If an HLA-DP incompatible transplant occurred, the decision to augment immunosuppression was made by a clinician at the time of transplantation or soon afterwards informed by further donor characterization and recipient antibody testing.

### Allograft Biopsies

Only “for cause” renal allograft biopsies were performed. Indications included delayed graft function, a sustained elevated urinary protein/creatinine ratio more than 50 mg/mmol, or a sustained rise in creatinine. C4d deposition was assessed using immunohistochemistry staining. Biopsies were scored using the Banff 2017 criteria.^18^ Biopsies receiving more than 1 Banff diagnosis (categories 2+3 or 2+4) were categorized as mixed rejection.

## Results

Between January 2013 and February 2020, 1355 adult kidney transplants were performed. The study included 114 patients (23 DPDSA, 18 DPnDSA, 27 HSP, and 46 Control) with a median follow-up of 1197 (range 1–2517) days. Throughout this period, 33 recipients had biopsy-proven rejection which encompassed ABMR, T-cell mediated rejection, borderline, and mixed rejection (15 DPDSA, 6 DPnDSA, 5 HSP, and 7 Control). Twenty-four patients had biopsy proven ABMR (15 DPDSA, 4 DPnDSA, 3 HSP, and 2 control). Twenty grafts failed (7 DPDSA, 6 DPnDSA, 4 HSP, and 3 Control).

### Patient Characteristics

The patient characteristics are shown in Table 1. HLA-DP antibodies were associated with increased sensitization as defined by cRF, and a higher proportion of patients had documented sensitizing events.

**Table 1:** Patient demographics

Variable	Total	DP-DSA	DP-nDSA	HSP	Control	*P*-value
Number	114 (100%)	23 (20%)	18 (16%)	27 (24%)	46 (40%)	
Age (yrs, SD)	46 (14)	43 (12)	46 (13)	45(14)	47 (15)	0.717^c^
Gender						0.002^a^
Male	60 (53%)	10 (44%)	8 (44%)	8 (30%)	34 (74%)	
Female	54 (48%)	13 (57%)	10 (56%)	19 (70%)	12 (26%)	
Primary renal disease						
DM/HTN	15 (13%)	3 (13%)	1 (6%)	3 (11%)	8 (17%)	
GN	25 (22%)	6 (26%)	7 (38%)	7 (26%)	5 (11%)	
Infection/Obstruction	19 (17%)	5 (22%)	2 (11%)	8 (30%)	4 (9%)	
Other	55 (48%)	9 (39%)	8 (44%)	9 (33%)	29 (63%)	
Median cRF (Q1–Q3)	66 (0–96)	94 (69–98)	84 (21–95)	95 (87–97)	0 (0)	<0.001^d^
cRF ≥ 85%	52 (46%)	16 (70%)	9 (50%)	27 (100%)	0	<0.001^a^
Sensitization history						
Blood transfusion	59 (52%)	19 (83%)	12 (67%)	18 (67%)	10 (22%)	<0.001^a^
Pregnancy	37 (32%)	9 (39%)	5 (28%)	14 (52%)	9 (20%)	0.067^a^
Previous transplant	47 (41%)	15 (65%)	10 (56%)	16 (59%)	6 (13%)	<0.001^a^
Pre-emptive	14 (12%)	3 (13%)	2 (11%)	0	9 (20%)	0.068^b^
Donor type						0.023^b^
DBD	68 (60%)	15(65%)	5 (28%)	20 (74%)	28 (61%)	
DCD	37 (33%)	7 (30%)	8 (44%)	6 (22%)	16 (35%)	
LD	9 (8%)	1 (4%)	5 (28%)	1 (4%)	2 (4%)	
Donor age (yrs, SD)	48 (16)	45 (16)	49 (22)	43 (12)	51 (17)	0.209^c^
HLA mismatch level						0.001^b^
1	19 (17%)	5 (22%)	3 (17%)	8 (30%)	3 (7%)	
2	28 (25%)	7 (30%)	1 (6%)	5 (19%)	15 (33%)	
3	57 (50%)	10 (44%)	7 (39%)	14 (52%)	26 (57%)	
4	10 (9%)	1 (4.3%)	7 (39%)	0	2 (4%)	
Graft number						<0.001^b^
1	70 (61%)	8 (35%)	10 (56%)	10 (37%)	42 (91%)	
2	36 (32%)	11 (48%)	6 (33%)	16 (59%)	3 (7%)	
3	7 (6%)	3 (13%)	2 (11%)	1 (4%)	1 (2%)	
4	1 (1%)	1 (4%)	0	0	0	
Median CIT (hrs, Q1–Q3)	14 (11–17)	17 (13–18)	13 (9–17)	15 (12–17)	13 (10–14)	0.002^d^
DGF	32 (28%)	10 (44%)	6 (33%)	11 (41%)	5 (11%)	0.007^a^
Induction agent						0.115^a^
Alemtuzumab	80 (70%)	15 (66%)	16 (89%)	21 (78%)	28 (61%)	
Basiliximab	34 (30%)	8 (35%)	2 (11%)	6 (22%)	18 (39%)	
Maintenance immunosuppression						
Tacrolimus	100%	100%	100%	100%	100%	
MMF	51 (45%)	20 (87%)	5 (28%)	8 (30%)	18 (39%)	<0.001^a^
Prednisolone	17 (15%)	10 (44%)	2 (11%)	3 (11%)	2 (4%)	<0.001^b^
Augmented immunosuppression	24 (21%)	17 (74%)	1 (6%)	4 (15%)	2 (4%)	<0.001^b^

CIT, cold ischemic time; cRF, calculated reaction frequency; DBD, donation after brain death; DCD, donation after cardiac death; DGF, delayed graft function; DM, diabetes mellitus; DSA, donor-specific antibodies; GN, glomerulonephritis; HSP, highly sensitized patients; HTN, hypertension; LD, live donor; MMF, mycophenolate mofetil.

Q1–25^th^ percentile, Q3–75^th^ percentile.

a Chi squared test.

b Fisher’s exact test.

c One way analysis of variance.

d Kruskall wallis test.

HLA mismatches were recorded using the United Kingdom NHSBT mismatch categories (Table 2).^19^ Three DPDSA patients received a kidney that was fully matched at the HLA-A HLA-B, HLA-C, HLA-DR and HLA-DQ loci. DPnDSA and control patients were more likely to receive a level 3 (A/B/DR 001, 011, 101, 111, 201, 211, 120, 020, 220) or 4 (A/B/DR 021, 121, 221, 002, 102, 202, 012, 112, 212, 022, 122, 222) mismatch kidney compared with the DPDSA and HSP groups. A higher proportion of DPDSA and HSP patients received a DBD graft compared with the DPnDSA and controls. HSP including DPDSA patients received grafts with a median cold ischemia time that was significantly greater than the remainder of the study cohort. These differences reflect the national allocation policy of the relevant era which prioritized HSPs to receive 000 mismatched DBD kidney grafts after pediatric recipients.

**Table 2:** NHSBT-ODT mismatch levels^19^

Level	Summary of mismatches at A,B and DR loci	A, B, DR mismatches included
1	000	000
2	0DR and 0/1 B	100, 010, 110, 200, 210
3	0DR and 2B or 1DR and 0/1B	001, 011, 101, 111, 201, 211, 120, 020, 220
4	1DR and 2B or 2DR	021, 121, 221, 002, 102, 202, 012, 112, 212, 022, 122, 222

Due to the presence of HLA-DP DSAs, DPDSA patients were more likely to be maintained on augmented immunosuppression at the time of transplant compared with the control groups. Four patients received prophylactic perioperative plasma exchange and intravenous immunoglobulin.

Forty-one patients had at least 1 HLA-DP antibody in their TOO sera, and 70% had antibodies against several HLA-DPB1 antigens (median number of specificities 10, interquartile range = 11). Twenty-three patients had 1 or more HLA DPB1-DSA, with a median cumulative MFI 11,009 (range 2141–47,349). Additional HLA-DPA1 DSAs could not be excluded in 5 DPDSA recipients due to the configuration of the HLA-DPA1 and HLA-DPB1 antigens within the microbead kits.

Twenty-one of the 23 DPDSA TOO sera were retested using Immucor kits and this confirmed HLA-DP DSAs in 16 of 21 samples. Two samples contained DP20 antibodies, which were not represented in the Immucor kit. The other 3 samples which tested negative using Immucor had a mean MFI of 2570. These results are demonstrated in Supplementary Table S1.

### Routine Laboratory Tests are Unable to Risk Stratify Transplants With Preformed HLA-DP Antibodies

#### Complement Dependent Cytotoxicity/FXM Testing

We attempted to risk stratify DPDSA patients using routine laboratory methods. The patient groups were compared with FXM results. Out of 95 “wet” crossmatches performed, 19 generated a positive BFXM result. A higher proportion of BFXM positivity occurred in patients with HLA-DP antibodies (DPDSA and DPnDSA groups) compared with patients who did not have HLA-DP antibodies (*P* = 0.0776, Chi Squared test, Table 3). In the 5 patients where HLA-DPA DSAs could not be excluded, a FXM was performed in 4 patients (BFXM positive in 2 of 4 and negative in 2 of 4 patients). One transplant proceeded following a virtual crossmatch (DPA1 DSA noted. Either DPA1^∗^02:01 average MFI 7126 or DPA1^∗^02:02 average MFI 6323). This multidisciplinary decision was made to minimize cold ischemia time with the local experience at the time that DPA1 antibodies were unlikely to cause FXM positivity.

**Table 3:** Comparison of B-cell flow crossmatch reactivity.

Group	FXM B negative	FXM B positive
DP-DSA (*n* = 22)	15 (68%)	7 (32%)
DP-non DSA (*n* = 18)	12 (67%)	6 (33%)
HSP (*n* = 24)	21 (88%)	3 (12%)
Control (*n* = 31)	28 (90%)	3 (10%)
Total (*N* = 95)	76 (80%)	19 (20%)

DSA, donor-specific antibodies; HSP, highly sensitized patients; FXM, flow crossmatch

The crossmatch results for 19 cases have been excluded (15 virtual crossmatches in the control group, 1 virtual crossmatch in the DP-DSA group, 1 inconclusive result in the DP-non DSA group, 2 missing crossmatch records in the DPnDSA group).

Two DPnDSA samples generated a T-cell positive FXM but T-cell negative CDC crossmatch result. These transplants were progressed because the concurrent serum samples did not contain HLA-DSAs that could be attributed to the reactivity. All other T-cell crossmatches (CDC and FXM) were negative. There was 1 CDC B-cell positive crossmatch in the DPDSA group which was attributed to the HLA-DP DSA. Fifteen transplants in the control group proceeded following a virtual crossmatch and were coded as “BFXM negative” for subsequent analyses.

**Figure 2:**
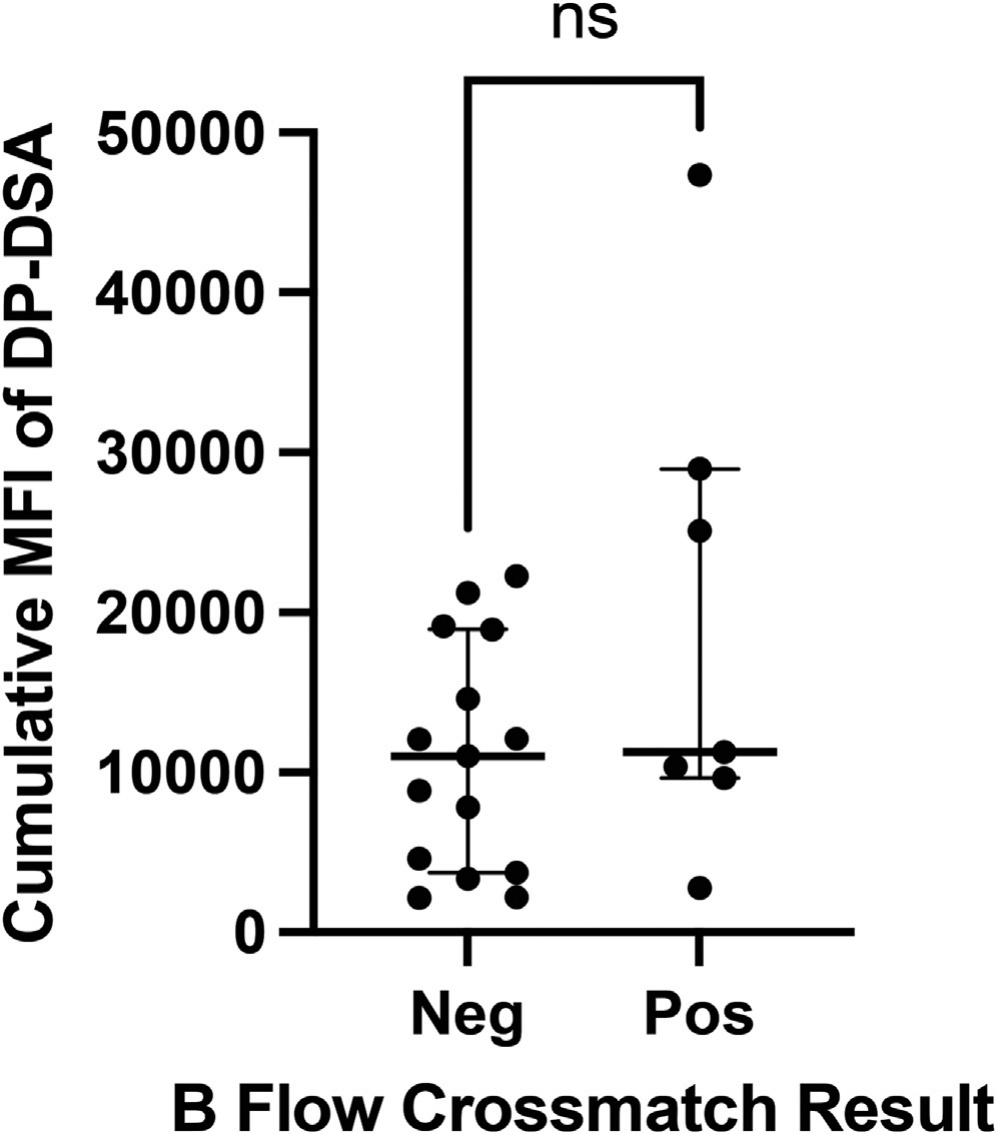
Scatter plots comparing the cumulative DP-DSA (MFI) with B flow crossmatch results. DSA, donor-specific antibodies; MFI, median fluorescent intensity. Individual results, median and interquartile range are shown.

We tested whether the TOO cumulative MFI was associated with an increased median channel fluorescence shift obtained from the BFXM. The values for cumulative MFI and median channel fluorescence were not correlated (R^2^ = 0.28) and a high cumulative MFI was not associated with BFXM positivity (BFXM negative median DP-DSA MFI 9931.5, range = 2141–22,252, BFXM positive median DP-DSA MFI 11,277, range = 2788–47,349, *P* = 0.2666, Figure 2).

We also considered the inferred donor HLA-DP antigen expression levels.^20^ Seventeen donors had high expression levels; 1 donor-recipient pair was removed from this analysis because only a virtual crossmatch had been performed. We obtained a positive crossmatch in 31% of cases where donors had high DP expression levels. The cumulative DSA associated with the positive crossmatch ranged from 2788 to 28,997 MFI. The negative crossmatches were associated with DSA MFIs ranging from 220 to 22,252. For cases with low expression (*n* = 6), we obtained a positive crossmatch in 33% of cases, with an associated DSA MFI of 10,350 to 47,439. Negative crossmatches were associated with DSAs ranging from 2141 to 19,136 MFI. This can be interpreted as no correlation *in vitro* between measured HLA-DP MFI and donor-specific reactivity measured by BFXM.

### HLA-DP Antibodies and Rejection Free Survival

**Figure 3:**
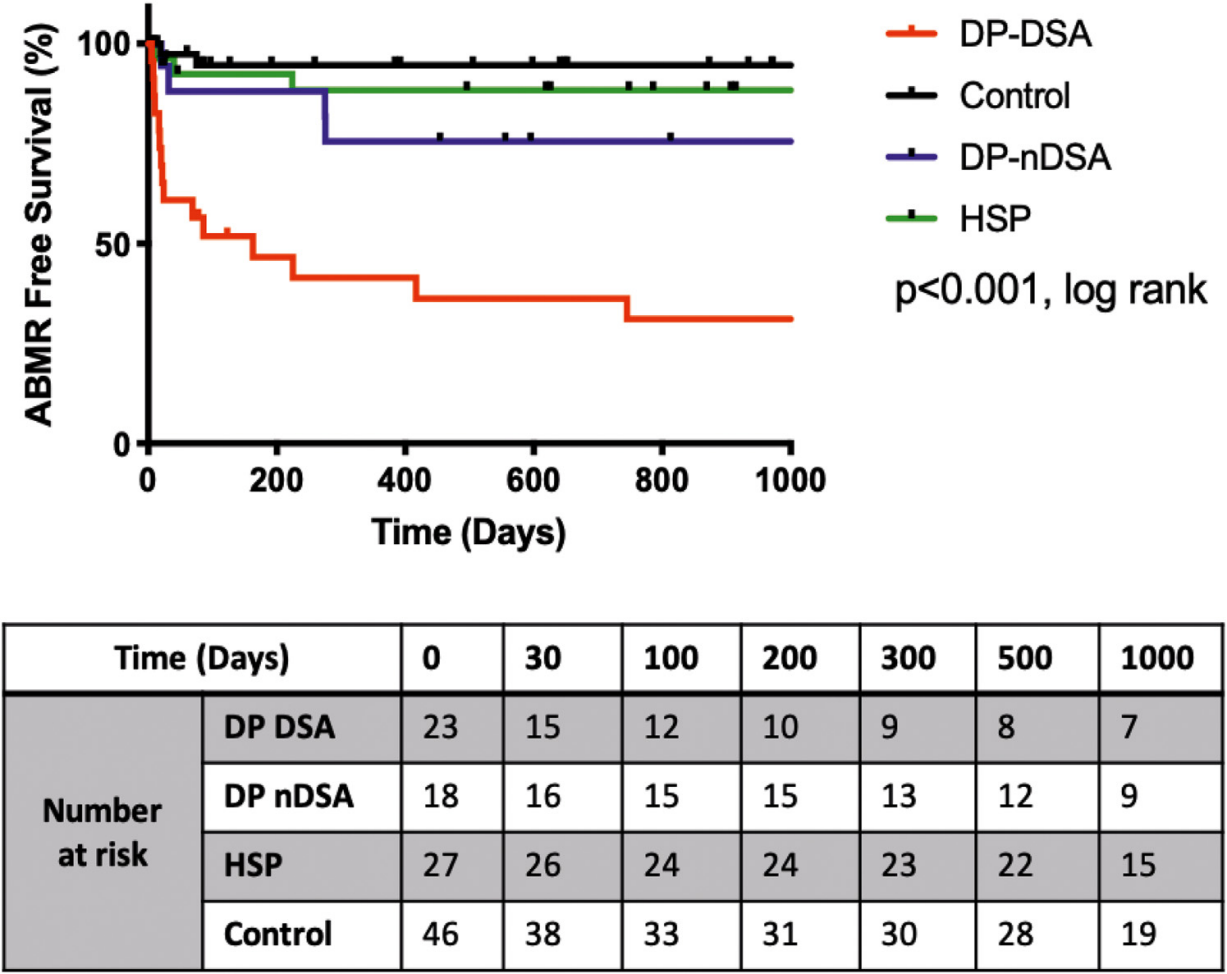
Kaplan-Meier curve demonstrating the ABMR-free survival up to 1000 days for patients within the DP-DSA, DP-nDSA, HSP and control groups. ABMR, antibody-mediated rejection; DSA, donor-specific antibodies; HSP, highly sensitized patients.

We then studied the relationship between the antibody profile and clinical episodes of ABMR. Throughout the follow-up period, 109 “for cause” biopsies were performed (33 DPDSA, 16 DPnDSA, 30 HSP, and 30 Control) and 24 patients had biopsy proven ABMR (15 DPDSA, 4 DPnDSA, 3 HSP, and 2 control). The Kaplan-Meier survival estimates for ABMR-free survival for the 4 cohorts is demonstrated in Figure 3. We observed that DPDSA patients had a significantly reduced ABMR-free survival compared with the control group (HR = 19.026, *P* < 0.001), with a median time to ABMR of 22 days. Univariate analyses using the Cox proportional hazards model found that patient group (DPDSA HR = 19, *P* < 0.001, DPnDSA HR = 4.54, *P* = 0.081), cRF more than 85% (HR = 3.37, *P* = 0.01), regrafts (HR = 2.76, *P* = 0.016) and BFXM positivity (HR = 3.655, *P* = 0.03) were associated with a reduced ABMR-free survival. These were entered into a multivariable model, and DPDSA remained the single variable that was associated with reduced ABMR-free survival (HR = 9.578, *P* = 0.012).

**Figure 4:**
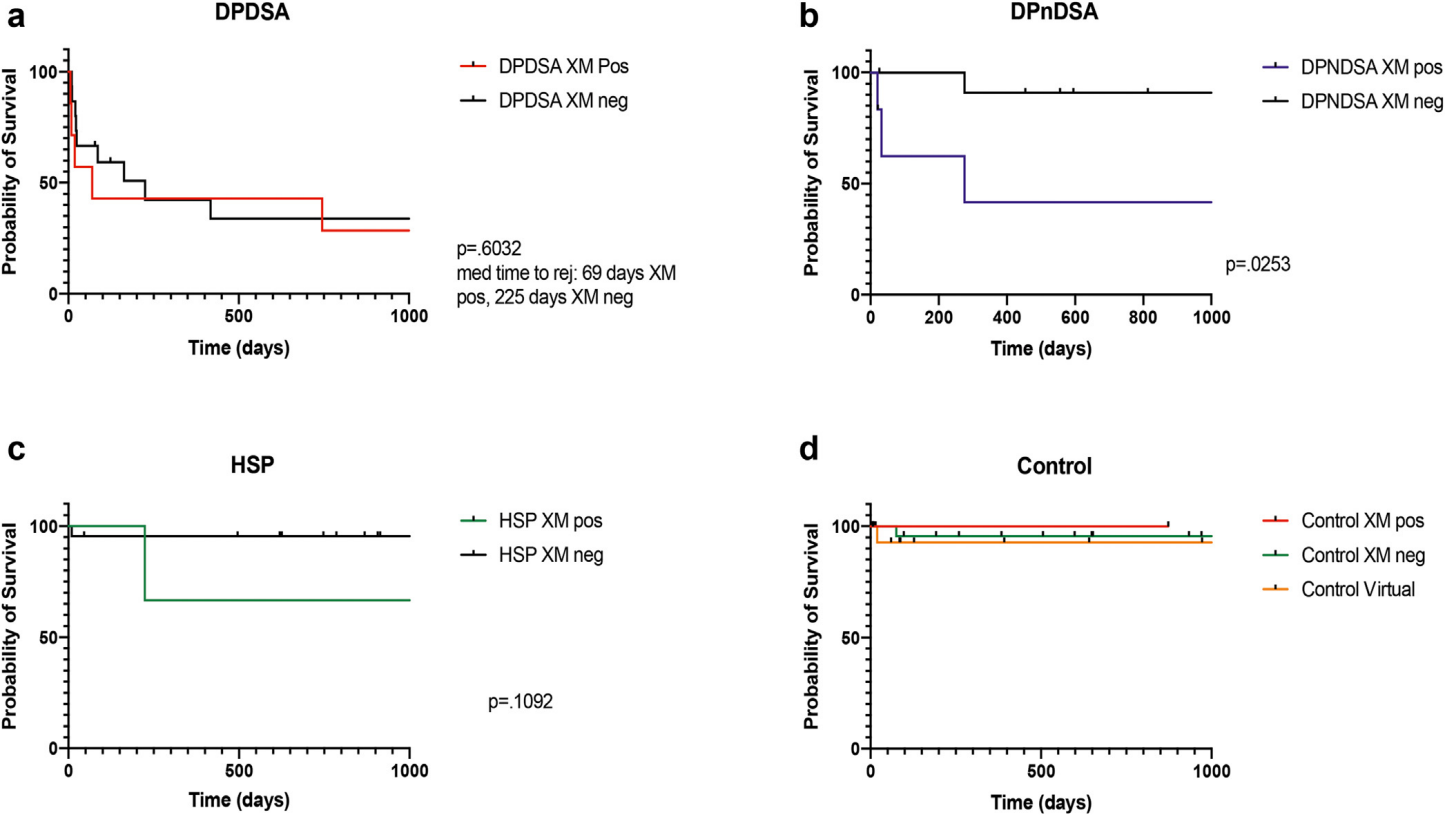
Kaplan-Meier curves demonstrating ABMR-free survival for the first 1000 days, stratified according to BFXM results in each of the 4 cohorts. (a) DPDSA (b) DPnDSA (c) HSP (d) Control. DSA, donor-specific antibodies; HSP, highly sensitized patients.

BFXM positivity was investigated further by constructing Kaplan-Meier curves for each cohort under study, comparing ABMR-free survival with BFXM positivity (Figure 4a-d). In the DPDSA and control groups, BFXM positivity was not associated with a significant difference in ABMR-free survival. There was a trend toward a reduced ABMR-free survival in BFXM positive HSP recipients, however this was not statistically significant (*P* = 0.192). Interestingly, a positive BFXM was associated with a reduced ABMR-free survival in the DPnDSA group (Figure 4b, median survival 276 days, HR = 8.483, *P* = 0.0253).

Similar models were constructed to investigate the variables associated with reduced overall rejection free survival (encompassing ABMR, T-cell mediated rejection, and mixed rejection). DPDSA was associated with an increased risk of rejection on univariate analysis (HR = 6.129, *P* < 0.001), however this was not statistically significant in the multivariable analysis (HR = 2.855, *P* = 0.093)

### Biopsy Results

**Figure 5:**
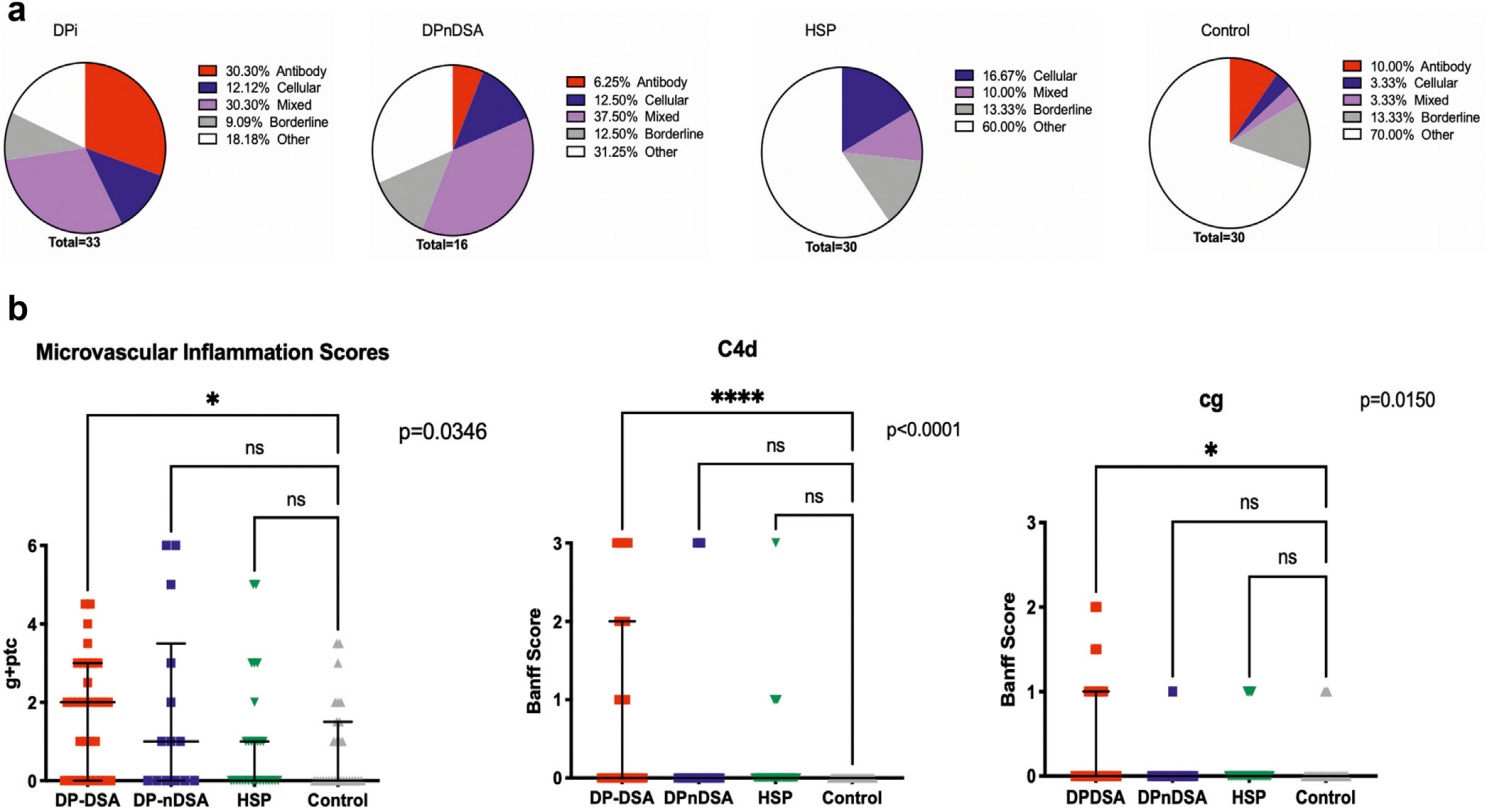
Analysis of Banff histological lesions from indication biopsies performed in each cohort. (a) Proportion of biopsy diagnoses (ABMR, TCMR, mixed rejection, borderline rejection or other). (b) Scatter plots demonstrating the individual scores, median and interquartile range for each Banff lesion. DSA, donor-specific antibodies; HSP, highly sensitized patients; TCMR, T-cell mediated rejection. The microvascular inflammation scores are the sum of g + ptc scores. Comparisons were made using the Kruskal-Wallis test.

Eighty-one percent of the DPDSA biopsies had rejection, of which ABMR and mixed rejection were the most common diagnoses. In the DPnDSA group, 68.75% of the biopsies were reported as rejection. Nevertheless, there was a higher proportion of mixed rejection (37.5%). Conversely, HSP and control patients were more likely to receive an alternative diagnosis (Figure 5a).

We assessed the indication biopsies for histological lesions that are associated with inferior clinical outcomes (Figure 5).^21^^,^^22^ The DPDSA biopsies were associated with higher microvascular inflammation (MVI) (*P* = 0.0346), higher C4d scores (*P* < 0.0001), and higher transplant glomerulopathy scores (*P* = 0.015) compared with the control patients (Figure 5b). There were higher cg scores in the DPDSA biopsies compared with the DPnDSA biopsies (mean rank difference = 16.58, *P* = 0.0384), however the difference in MVI scores were not statistically significant. Interestingly, less tubular atrophy was found in the DPDSA (mean rank difference = 18.02, *P* = 0.0331) and DPnDSA (mean rank difference = 21.57, *P* = 0.0439) patients and less fibrosis in the DPDSA patients (mean rank difference = 21.61, *P* = 0.0174) when compared with controls. This could not be explained by donor age or median time to biopsy, which was similar across the groups (DPDSA = 69 days, interquartile range = 207.5, DPnDSA = 157 days, interquartile range = 394.55, control = 143 days, interquartile range = 433.25, *P* = 0.3615).

The DSA profiles obtained from the DPDSA group over the period of follow-up, together with initial post-transplant management and subsequent clinical outcomes are depicted in Supplementary Table S1.

### Graft Survival

**Figure 6:**
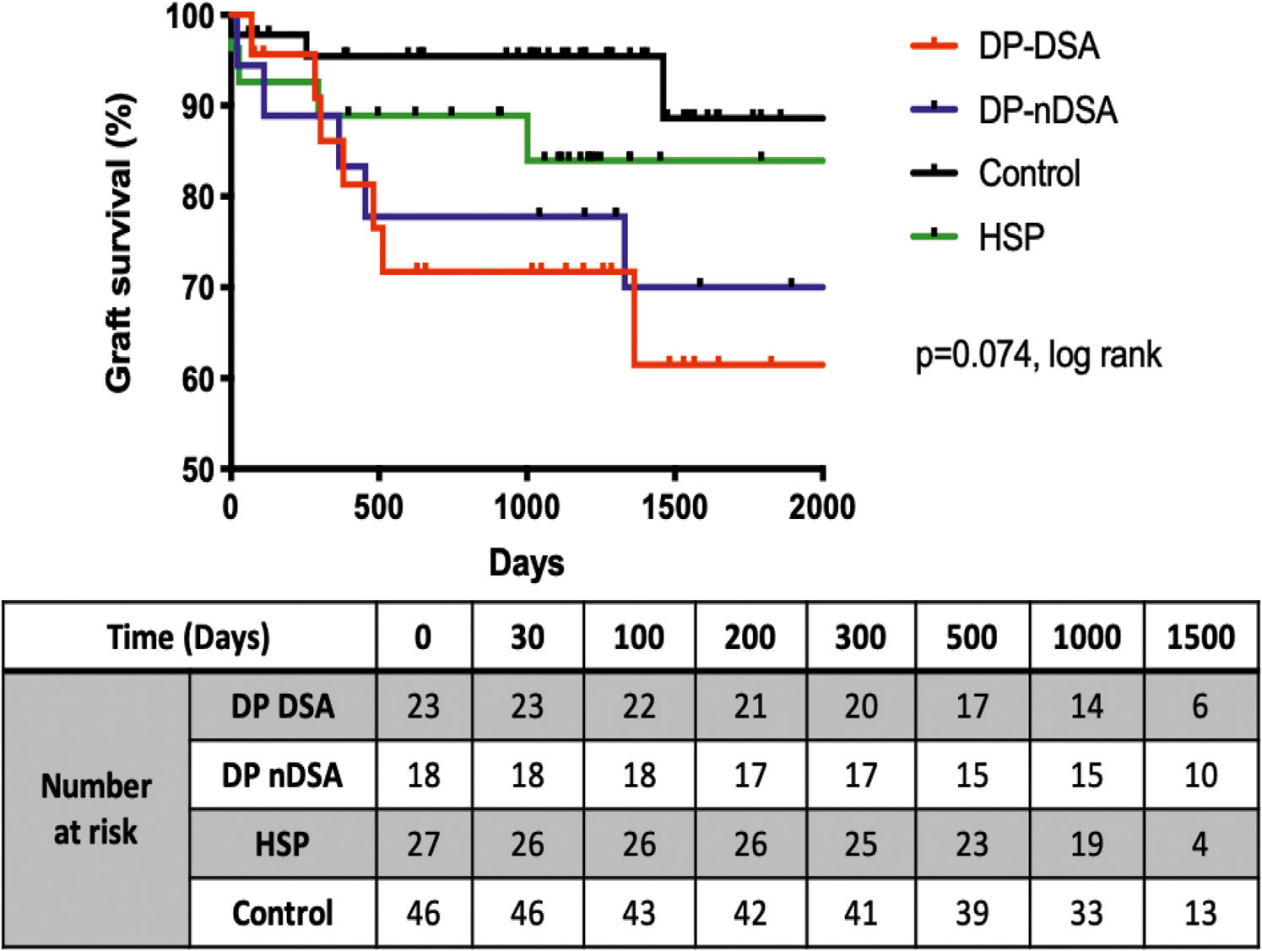
Kaplan-Meier survival curves depicting the estimated death censored Graft Survival in the first 2000 days for the patients within the DP-DSA, DP-nDSA, HSP and control groups. DSA, donor-specific antibodies; HSP, highly sensitized patients.

Having established that HLA-DP antibodies were associated with reduced ABMR-free survival, we asked if they were associated with reduced graft survival. The Kaplan-Meier curves comparing death-censored graft survival across the 4 groups are depicted in Figure 6. Univariate analyses using the Cox proportional hazards model identified 4 variables associated with reduced graft survival, which included DPDSA (HR = 5.218, *P* = 0.048), delayed graft function (HR = 3.376, *P* = 0.016), regraft (HR = 7.461, *P* = 0.002), and high calcineurin inhibitor variability^23^^,^^24^ more than 3 months post-transplant (HR = 9.505, *P* < 0.001). In the multivariable analysis, DP-DSA was not associated with graft loss. Regrafts remained the single independent variable for reduced graft survival (HR = 5.135, *P* = 0.028). The documented causes of graft loss in the DPDSA group *(n =* 7) included ABMR with ischemia-reperfusion injury, chronic ABMR, recurrent focal segmental glomerulosclerosis, BK nephropathy, cytomegalovirus disease with evidence of chronic ABMR, and chronic allograft nephropathy (*n* = 2).

The median estimated glomerular filteration rate of the whole cohort was 40, 45 and 42 ml/min per 1.73m^2^ for 3 months, 1 year and 3 years post-transplant, with no statistically significant difference across the groups. For the purposes of this assessment, if a graft failed, the estimated glomerular filteration rate was coded as 5 ml/min per 1.73m^2^.

### Epitope Analysis

We attempted to further risk-stratify DPDSA transplants by determining the antigenicity of DPB1 mismatched epitopes. HLAMatchmaker and https://epregistry.com.br were used to determine any “exposed antibody-verified” DPB1 epitope mismatches that corresponded with the recipient DSA profile.^25^ There was a trend toward reduced but nonstatistically significant ABMR-free survival (HR = 1.867, *P* = 0.3308) and graft survival (HR = 2.979, *P* = 0.2880) in donor-recipient pairs with an 84 DEAV mismatch. In addition, a 96R mismatch was associated with reduced ABMR-free survival (HR = 10.47, *P* = 0.0040) but a trend toward improved graft survival (HR = 0.33, *P* = 0.4435). It was difficult to draw firm conclusions due to the small patient numbers and multiple epitope mismatches within each donor-recipient pair.

Classification of permissive and nonpermissive donor and recipient pairs using the T-cell epitope algorithm did not risk stratify DPDSA transplants.^26^

### Donor HLA-DPB1 Expression Levels

Finally, DPDSA patients were categorized into low and high expression groups inferred from the donor HLA-DPB1 alleles.^15^^,^^16^ Donor HLA-DPB1 expression levels were not associated with rejection free survival, however there was a trend toward reduced graft survival in high donor HLA-DPB1 expressors, (HR = 2.505, *P* = 0.3578).

## Discussion

Chronic ABMR is a major cause of renal allograft loss and it is strongly linked to the development of donor-specific HLA class II antibodies.^4^^,^^27^ Class II DSAs are associated with the process of transplant glomerulopathy leading to interstitial fibrosis, tubular atrophy, and eventual graft failure.^28^ A primary site of allorecognition is the donor endothelium and though HLA-DR expression is usually higher in the resting state, the expression of HLA-DQ and DP antigens are induced by inflammatory stimuli such as rejection or ischemia-reperfusion injury, possibly mediated by γ-interferon.^29^

The HLA-DP antigen consists of a heterodimer of 2 peptide chains DPα and DPβ, which are derived from the polymorphic DPA1 and DPB1 genes respectively. Population genetic studies have revealed strong linkage disequilibrium between DPA1 and DPB1 but only weak linkage with HLA-DR and HLA-DQ.^30^ Consequently, there is an 80% chance of a DP mismatch even if an unrelated donor and recipient pair are fully matched at the A, B, C, DR and DQ loci. Initial experiments in mixed lymphocyte reactions revealed inconsistent results between different DP types, limiting its utility in clinical practice.^31^ With the advent of molecular typing, it became clear that substantial polymorphism exists within the DPB1 gene.^32^ Furthermore, associations have been discovered relating to allelic variation and expression levels in both autoimmunity and the development of GvHD in stem cell transplantation.^15^^,^^33^

In the United Kingdom, HLA-DP antibodies have not been used historically to define unacceptable antigens in the national deceased donor kidney allocation scheme. The introduction of solid phase assays for HLA antibody detection and readily available molecular typing methods has led to a reappraisal of the role of HLA-DP in renal transplantation.^34^ This study was undertaken to address this situation and to try to guide the clinician when faced with DP-DSAs particularly because these may become apparent after the renal transplant has occurred.

An early registry-based study found no relationship between DP mismatch and outcomes in first transplants.^5^ Nevertheless there was a deleterious effect on graft survival in subsequent grafts especially in recipients with cRF greater than 50%. A European study of 291 patients showed that HLA-DP antibodies were common, present in nearly half of recipients with DSAs.^35^ Whereas the presence of class II DSAs was associated with poorer graft survival, there was no additive effect of HLA-DP antibodies. Other studies have reported deleterious effects, but the DP-DSAs were usually present with other HLA-DSAs making it difficult to disentangle specific effects of the HLA-DP antibodies.^36^ There have also been case reports suggesting that isolated DP antibodies can mediate significant graft damage with ABMR and early graft loss implying that such antibodies may be directly pathogenic.^8^^,^^10^^,^^37^ A French study reported 26 patients with HLA-DP DSAs and demonstrated an association with a significantly increased risk of a positive FXM, ABMR, and graft loss compared to unsensitized controls. This risk was similar to recipients with DSAs against HLA-A, HLA-B, HLA-DR, and HLA-DQ.^38^ A recent retrospective study identified 13 patients with pre-existing isolated HLA-DP DSAs, 6 of whom experienced ABMR and 3 lost their grafts.^39^

There is good evidence that there is a phenotypic difference between pre-existing HLA-DSAs and *de novo* DSAs. *De novo* antibodies tend to be HLA class II antibodies and are associated with more chronic damage at the time of biopsy with worse clinical outcome.^40^ Against this background we assessed the effect of isolated pre-existing HLA-DP DSAs in our patient population over a 7-year period. During this time, donor HLA-DP typing was not routinely performed therefore transplants would proceed in the setting of a negative crossmatch, and knowledge of pre-existing HLA-DP DSAs often only became apparent following transplantation. This was not surprising because recent evidence suggests that HLA-DP antibody levels with MFIs less than 10,000 are associated with a negative CDC crossmatch and even above 10,000, only 70% will register as positive.^39^ This may be due to the lower expression levels of HLA-DP antigens compared with other human leukocyte antigens on resting cells.^41^

In this study, we compared kidney transplant recipients with isolated pre-existing HLA-DP DSAs with 2 other sensitized groups (DPnDSA and HSP) and a third control group. Unsurprisingly, the 3 sensitized groups included more females and sensitizing events especially blood transfusion and previous transplantation. There were differences in donor type with only 1 living donor in the DPDSA group. The UK kidney allocation system prioritizes implantation of sensitized and long-waiting patients over geographical proximity. Transplants therefore tended to be better matched in the sensitized patients where there was a high proportion of regrafts (65% of the DPDSA group and 63% of the HSP group). Cold ischemia times were also longer in the sensitized patients, and this may partially explain the associated increased rates of delayed graft function, although alloimmune mechanisms may also be operating. For example, the longer cold ischemia time may have led to increased ischemia-reperfusion injury with upregulation of HLA-DP expression.

The presence of DP-DSAs often were reported following the transplant and the clinicians usually commenced mycophenolate mofetil, prednisolone, or both. As a result, augmented immunosuppression was used in 74% of patients with DP-DSAs. This was not seen in the HSP population because patients who received transplants following delisting or desensitizing strategies were excluded from this study.

In this study, 32% of DPDSA patients had a positive B-cell crossmatch which was increased compared to other groups but not significantly so. There was no correlation between the measured HLA-DP antibodies in the DPDSA group and the total donor-specific reactivity as measured by the BFXM. This is consistent with previous data describing a negative CDC crossmatch in patients with DP-DSA levels less than 10,000 MFI, and approximately 30% of those with MFI greater than 10,000.^39^ Nevertheless, DP-DSAs were associated with significant episodes of ABMR with more than half (15 of 23) suffering from ABMR (median time to ABMR 22 days, twenty-fifth centile 14, seventy-fifth centile 125 days). After multivariate analysis, HLA-DP-DSA was the single factor that was associated with the development of clinical ABMR (HR = 9.6). Though it does not prove causality this supports the observations of others that TOO HLA-DP DSAs are associated with significant clinical events.^8^^,^^9^ Moreover, in our cohort the BFXM did not add any further information. Mechanistically, this raises the question as to whether HLA-DP DSAs are directly pathogenic or simply a marker of an increased immunoreactive phenotype, and this is a hypothesis that warrants further study. Recent observations that HLA-DQ DSAs can bind to the donor endothelium and modulate the generation of T-regulatory cells support possible indirect mechanisms.^42^

DPDSA renal transplant biopsies did show evidence of increased MVI, C4d deposition, and transplant glomerulopathy although we acknowledge that in the absence of protocol biopsies there may have been a lower threshold to perform biopsies in this group. This did translate into a trend toward lower graft survival in the DPDSA group although this did not reach statistical significance, possibly due to low numbers overall. There was also no association with graft function or proteinuria and a larger series will be required to address this.

We did evaluate certain high-risk mutations, such as 84 DEAV mismatch, but had insufficient numbers to draw valid conclusions.^39^ Similarly, we were unable to demonstrate a significant association with the inferred donor HLA-DPB1 expression levels that have been described in stem cell transplantation.^15^

There is increasing evidence of processing artifacts associated with the production of Luminex microbead arrays, which may result in false positivity, especially among the class II HLA.^43^ We attempted to address this using assays from 2 different manufacturers. We obtained a consensus in 16 of 21 TOO samples tested. Two samples were not in agreement due to differing antibody specificities included in the assay kits. In the remaining 3 samples, DPDSAs were detected using the ONELAMBDA, but not the Immucor assay. The overall “strength” of these DSAs were relatively low, and inconsistencies could be explained by differing assay sensitivities as a result of varying antigen densities in the presence of low-level antibodies, or by the conformational changes of antigens found on the different bead kits. Although these 3 patients did not lose their grafts during the follow-up period, 1 did exhibit early ABMR, therefore further investigation is required to test the clinical utility of using a combination of bead kits for risk stratification in the presence of HLA-DP antibodies.

We noted a 10% rate of ABMR in the control (standard immunological risk) group, however there was no ABMR in the HSP group. Whereas the HSPs would have undergone a detailed longitudinal characterization of their HLA antibody profile over a prolonged wait time, the control group would not have been as closely scrutinized. In addition, all patients are routinely screened for HLA antibodies using the LABScreen mixed bead test, with a reflex for further characterization using ONELAMBDA SABs if positive. It is possible that samples test negative using the mixed screen, yet are positive on testing with SAB. Unfortunately, testing all samples from every wait listed patient with SAB is cost prohibitive, and we acknowledge that there may be the rare case where a patient who screened negative in the control group may have an uncharacterized DSA.

There is currently no consensus method for calculating the antibody “strength” when a panel includes more than 1 bead per antigen or allele specificity. Our practice is to calculate the average MFI over all beads, unless there is a clear allelic antibody, at which point the MFI for the specific allelic antibody is reported, which can underestimate the amount of antibody present. Alternatively, adding the MFI obtained from each bead can lead to the overestimation of the antibody amount.

Non-HLA antibodies, which may have contributed to BFXM positivity in the DPnDSA cohort in the absence of measurable HLA-DSAs, were not investigated, and their role could not be excluded in the transplant outcomes.^44^

In summary we describe a cohort of patients who received a kidney transplant with pre-existing HLA-DP DSAs. We show that despite augmented immunosuppression, approximately half of these cases suffered from biopsy proven ABMR within the first 6 months that was not further informed by the FXM. This rejection was associated with increased histological damage and a trend toward worse graft survival. We suggest that kidney transplant recipients with pre-existing DP-DSA be considered a high-risk immunological group and are subjected to close monitoring in the first 6 months after transplantation.

## Disclosure

All the authors declared no competing interests.

## Supplementary Material

Supplementary File (PDF)

**Figure S1.** The definition of calculated reaction frequency, cRF.

**Figure S2.** The formulas for determining the threshold of positivity for HLA antibody detection using ONELAMBDA single antigen beads.

**Table S1.** Graphs demonstrating the kinetics of the donor-specific antibodies identified for each DPDSA patient over the period of follow up.
