# Supplementary material for: Isolated Pre-existing HLA-DP Donor-Specific Antibodies are Associated With Poorer Outcomes in Renal Transplantation
Source: Kidney Int Rep. 2022 Aug 3;7(10):2251–63. doi: 10.1016/j.ekir.2022.07.014 (PMC9546735; doi:10.1016/j.ekir.2022.07.014)
Supplement: Supplementary File (PDF) [file mmc1.pdf]

Supplementary Material

Supplementary Figures

$$A)\ normalised\ MFI = S\#N - (BG\#N + (SNC\ bead - BGNC\ bead))$$

$$B)\ NBG\ ratio = \frac{S\#N\ /SNC\ bead}{BG\#\ /BGNC\ bead}$$

Figure S 1. The formulae for determining the threshold of positivity for HLA antibody detection using ONELAMBDA single antigen beads. A) Formula for calculating the normalised median fluorescence intensity value for each HLA-coated bead. B) Formula for calculating the normalised background ratio to indicate the strength of the reaction above the negative control bead. S#N – sample specific fluorescent value for bead #N, SNC bead – sample-specific fluorescent value for the negative control bead, BG#N – background negative control (NC) serum fluorescent value for #N, BGNC bead – background NC serum fluorescent value for negative control bead

<u>Calculated Reaction Frequency (cRF)</u>

This estimates the breadth of HLA sensitisation demonstrated in a patient and is similar in principle to the US Organ Procurement and Transplantation Network CPRA (calculated panel reactive antibodies) tool. The calculation is performed using a tool provided by NHS-BT. The antibodies defined by the single antigen bead tests are put into the calculator together with the recipient's blood group. The tool compares the antibody profile with the frequency of HLA antigens that were present in the last 10000 blood group compatible transplants that were performed. The result refers to the proportion of the recent donor pool that were incompatible with the patient.

For example, a cRF of 85% can be interpreted as the patient is incompatible with HLA alleles that are present in 85% of the last 10000 blood group compatible donors.

Patients with a cRF $\geq$ 85% have traditionally been considered as 'highly sensitised patients' (HSPs).

This tool does not consider HLA-DPA1, -DPB1, or -DQA1 antibodies in the calculation.

Figure S2. Definition of calculated reaction frequency.[1]

Patient	HLA-DP DSA Present using ONELAMBDA and Immucor microbead kits?	Immunosuppression Plan at time of transplant	ABMR (Days)	Graft Loss (Days)
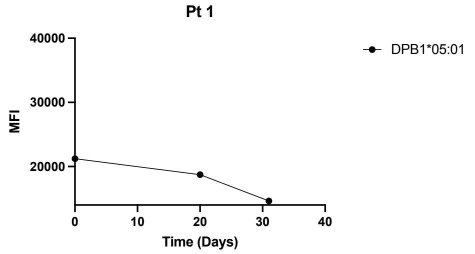	Unable to retest	Basiliximab Tacrolimus, MMF, Prednisolone	Yes (24)	Yes (69)
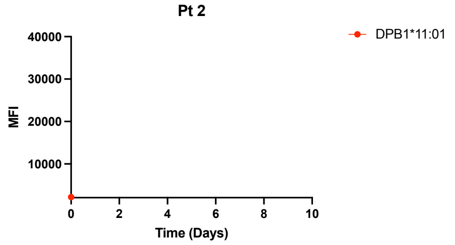	No	Alemtuzumab Tacrolimus, MMF	No	No
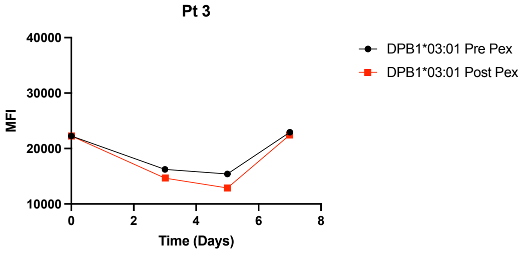	Yes	Pre-emptive plasma exchange Alemtuzumab Tacrolimus, MMF, Prednisolone	Yes (20)	No
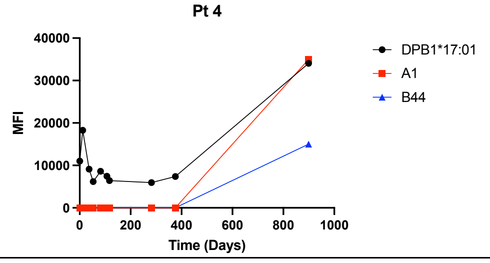	Yes	Basiliximab Tacrolimus, MMF	Yes (10)	Yes (482)
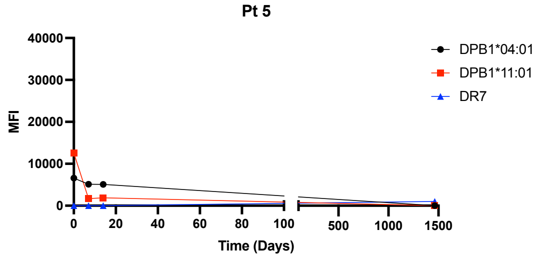	Yes	Basiliximab Tacrolimus, MMF, Prednisolone	No	No
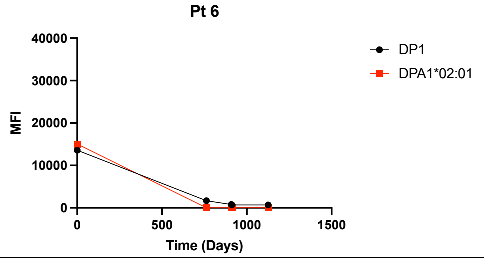	Yes	Alemtuzumab Tacrolimus, MMF	No	Yes (1363)

Patient	HLA-DP DSA Present using ONELAMBDA and Immucor microbead kits?	Immunosuppression Plan at time of transplant	ABMR (Days)	Graft Loss (Days)
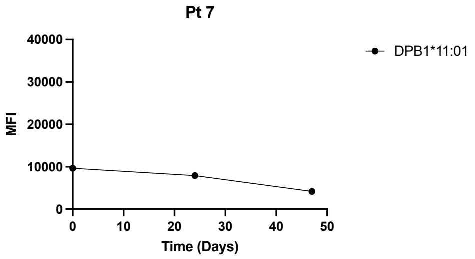	Yes	Basiliximab<br>Plasma Exchange<br>Tacrolimus, MMF,<br>Prednisolone	Yes<br>(18)	Yes<br>(513)
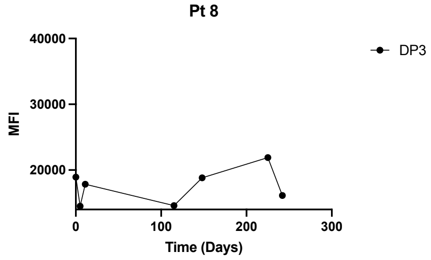	Yes	Alemtuzumab<br>Tacrolimus	Yes<br>(9)	Yes<br>(284)
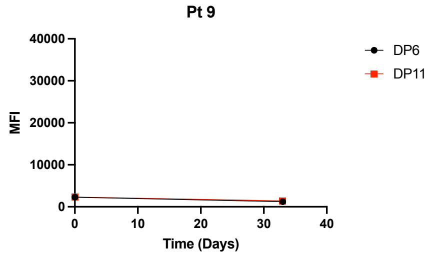	Unable to retest	Basiliximab<br>Tacrolimus, MMF	No	No
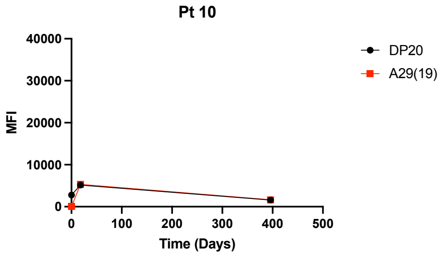	No – DP20 not present in LifeCodes kit	Basiliximab<br>Tacrolimus, MMF	No	No
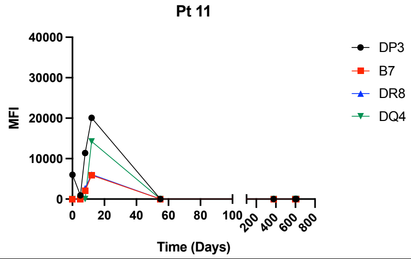	Yes	Alemtuzumab<br>Tacrolimus, MMF	Yes<br>(22)	No
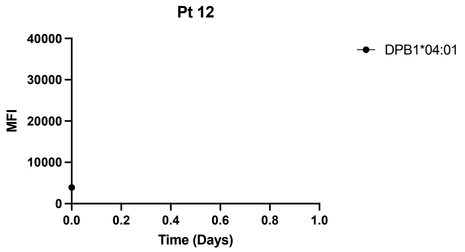	Yes	Basiliximab<br>Tacrolimus, MMF,<br>Prednisolone	No	No

Patient	HLA-DP DSA Present using ONELAMBDA and Immucor microbead kits?	Immunosuppression Plan at time of transplant	ABMR (Days)	Graft Loss (Days)
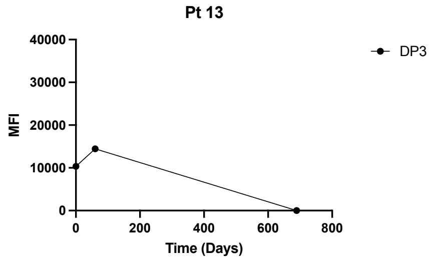	Yes	Alemtuzumab<br>Tacrolimus, MMF	Yes<br>(745)	No
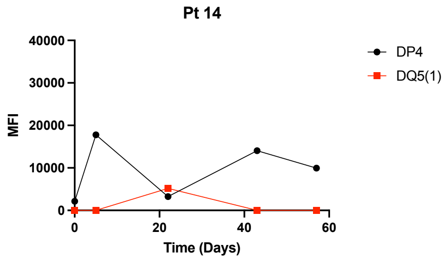	No	Alemtuzumab<br>Tacrolimus	Yes<br>(9)	No
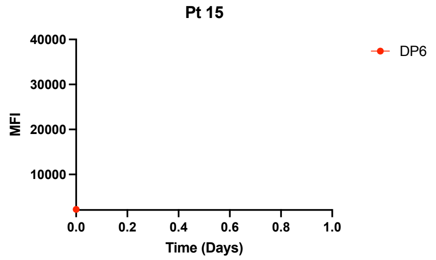	No	Alemtuzumab<br>Tacrolimus	No	No
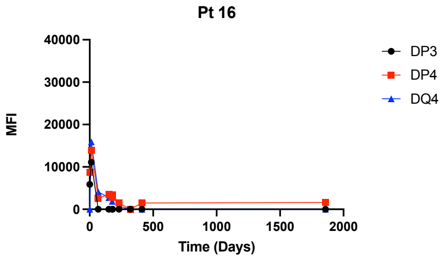	Yes	Basiliximab<br>Plasma Exchange<br>Tacrolimus, MMF,<br>Prednisolone	Yes<br>(170)	No
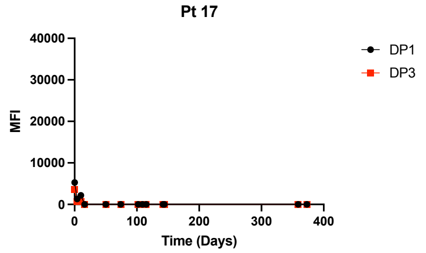	Yes	Alemtuzumab<br>Tacrolimus, MMF,<br>Prednisolone	Yes<br>(163)	Yes<br>(379)
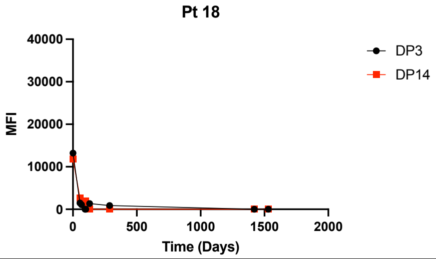	Yes	Alemtuzumab<br>Plasma Exchange<br>Tacrolimus, MMF,<br>Prednisolone	Yes<br>(69)	No

Patient	HLA-DP DSA Present using ONELAMBDA and Immucor microbead kits?	Immunosuppression Plan at time of transplant	ABMR (Days)	Graft Loss (Days)
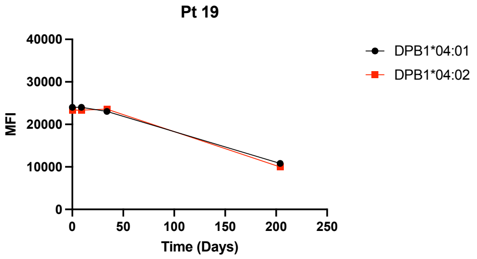	Yes	Alemtuzumab Tacrolimus, MMF, Prednisolone	Yes (5)	No
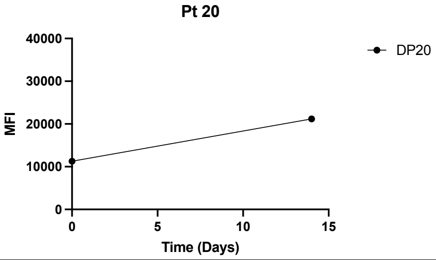	No - DP20 not present in LifeCodes Kit	Alemtuzumab Tacrolimus, MMF	Yes (8)	No
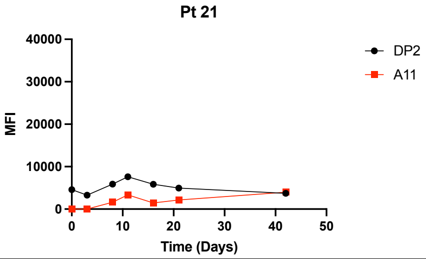	Yes	Alemtuzumab Tacrolimus, MMF, Prednisolone	No	No
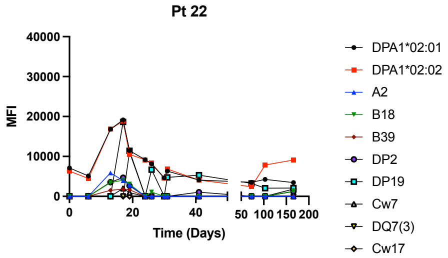	Yes	Alemtuzumab Tacrolimus, MMF	Yes (17)	No
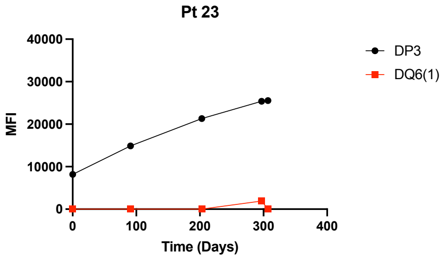	Yes	Alemtuzumab Tacrolimus, MMF	Yes (86)	Yes (303)

Table S1. Graphs demonstrating the kinetics of the donor specific antibodies for each DPDSA patient over the period of follow up. Clinical outcomes (including ABMR and Graft loss) are included. MFI = median fluorescent intensity. Samples were tested in real-time therefore the uncertainty of measurement associated with determining serial MFI values could not be controlled for. Routine/real-time sera were testing using ONELAMBDA SAB kits. The TOO sample was retested using Immucor Kits

References

1. Calculators - ODT Clinical - NHS Blood and Transplant. https://www.odt.nhs.uk/transplantation/tools-policies-and-guidance/calculators/. Accessed April 4, 2022.
